# Management of internal root resorption in the maxillary central incisor with fractured root using Biodentine

**DOI:** 10.1002/ccr3.4502

**Published:** 2021-07-21

**Authors:** Parisa Sanaei‐rad, Marjan Bolbolian, Faranak Nouri, Ehsan Momeni

**Affiliations:** ^1^ Department of Endodontics School of Dentistry Arak University of Medical Sciences Arak Iran; ^2^ Department of Endodontics School of Dentistry Qazvin University of Medical Sciences Qazvin Iran; ^3^ Department of Endodontics School of Dentistry Tehran University of Medical Sciences Tehran Iran; ^4^ Department of Oral medicine School of Dentistry Arak University of Medical Sciences Arak Iran

**Keywords:** apical abscess, biodentine, CBCT, internal root resorption

## Abstract

Biodentine is desirable to successfully manage internal root resorption, and the MTA apical plug backfilled with thermoplasticized gutta‐percha is suggested for the tooth with external apical root resorption.

## Graphical abstract

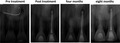

## 1 INTRODUCTION

This paper reports the successful management of internal root resorption (IRR) in the maxillary right central incisor with Biodentine. The maxillary left central incisor with chronic apical abscess was treated using MTA and thermoplasticized gutta‐percha. After 3 months, successful healing and resolution of signs and symptoms were observed.

Root resorption has been described as the loss of dentin as a result of odontoclastic action. It may be a physiologic process, which can be seen in deciduous teeth or occurred due to pathologic factors such as trauma and pulpal inflammation. Root resorption is categorized into internal or external resorption based on the location of the defect in regard to the root surface. The prevalence of internal root resorption (IRR) is very low, and it may be treated inappropriately due to the wrong diagnosis. IRR is occurred when the odontoclasts start to destruct the exposed mineralized dentin under the damaged predentin and protective odontoblasts. The progression of resorption depends on the bacterial stimulation of clastic cells responsible for hard tissue destruction. IRR is commonly seen as a radiolucent area around the pulpal cavity within the dentinal wall of incisors and mandibular molars, and it is usually asymptomatic.^1^, ^2^ Cone beam computed tomography (CBCT) is very efficient to improve diagnosis and/or management of complex endodontic problems. Due to the limitations of conventional radiography, CBCT is desirable for better and accurate diagnosis of periapical lesions, apical periodontitis, traumatic dental injuries, root resorptions, fractures, and root anatomy.^3^ IRR can be diagnosed by the visual examination based on tooth discoloration, conventional radiography, and CBCT. If left untreated, the pulp tissue will become necrotic and the infection will progress and may result in apical periodontitis.^4^ For the treatment of IRR, root canal treatment (RCT) is necessary when the tooth can be saved or the prognosis is favorable. Like any tooth with infected pulp, RCT is performed to remove the intraradicular bacteria and disinfect the root canal space. In IRR cases, the root canal filling material must be flowable in order to adequately fill the irregular defects resulting from internal resorption. In such conditions, thermoplasticized gutta‐percha (GP) has been generally used due to its flowability. In a case with the risk of perforation, the apical part of the root canal and the resorption area is filled with GP and a biocompatible material such as mineral trioxide aggregate (MTA), respectively.^5^, ^6^ Recently, Biodentine has been introduced as a new calcium silicate‐based material for filling resorptive defects. In addition to biocompatibility, Biodentine has some additional and desired properties over MTA, such as better antibacterial characteristics, bioactivity‐inducing hard tissue formation, good handling, self‐adhesion to dentine, no shrinkage, and a shorter setting time.^7^, ^8^ In the present case, the IRR was successfully managed in the maxillary right central incisor of a male patient using Biodentine. Simultaneously, the maxillary left central incisor with chronic apical abscess was obturated using MTA backfilled with Beefill thermoplasticized gutta‐percha. After 3 months, successful healing and resolution of signs and symptoms were observed.

## 2 CASE REPORT

**FIGURE 1:**
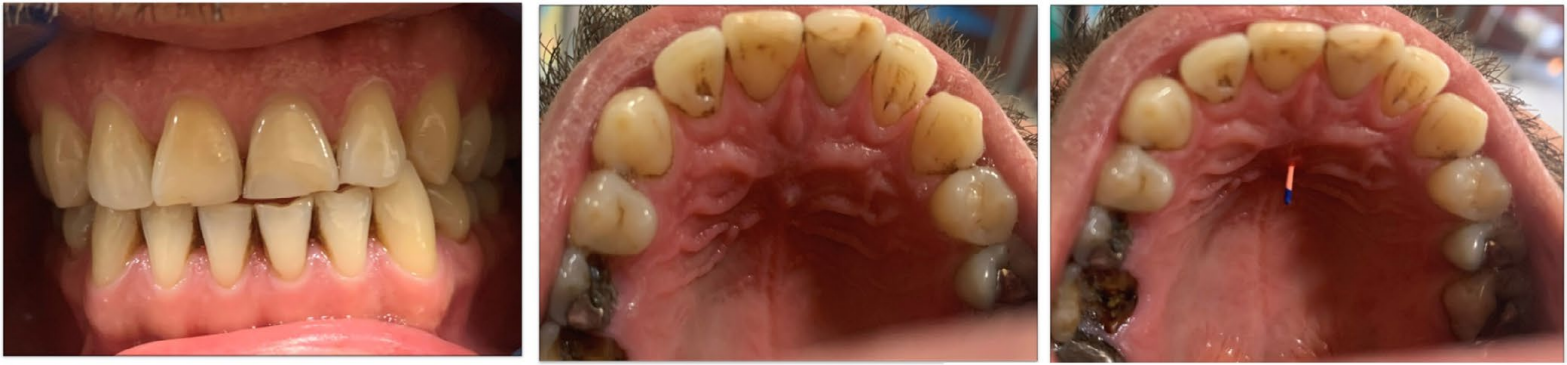
Intraoral photographs

**FIGURE 2:**
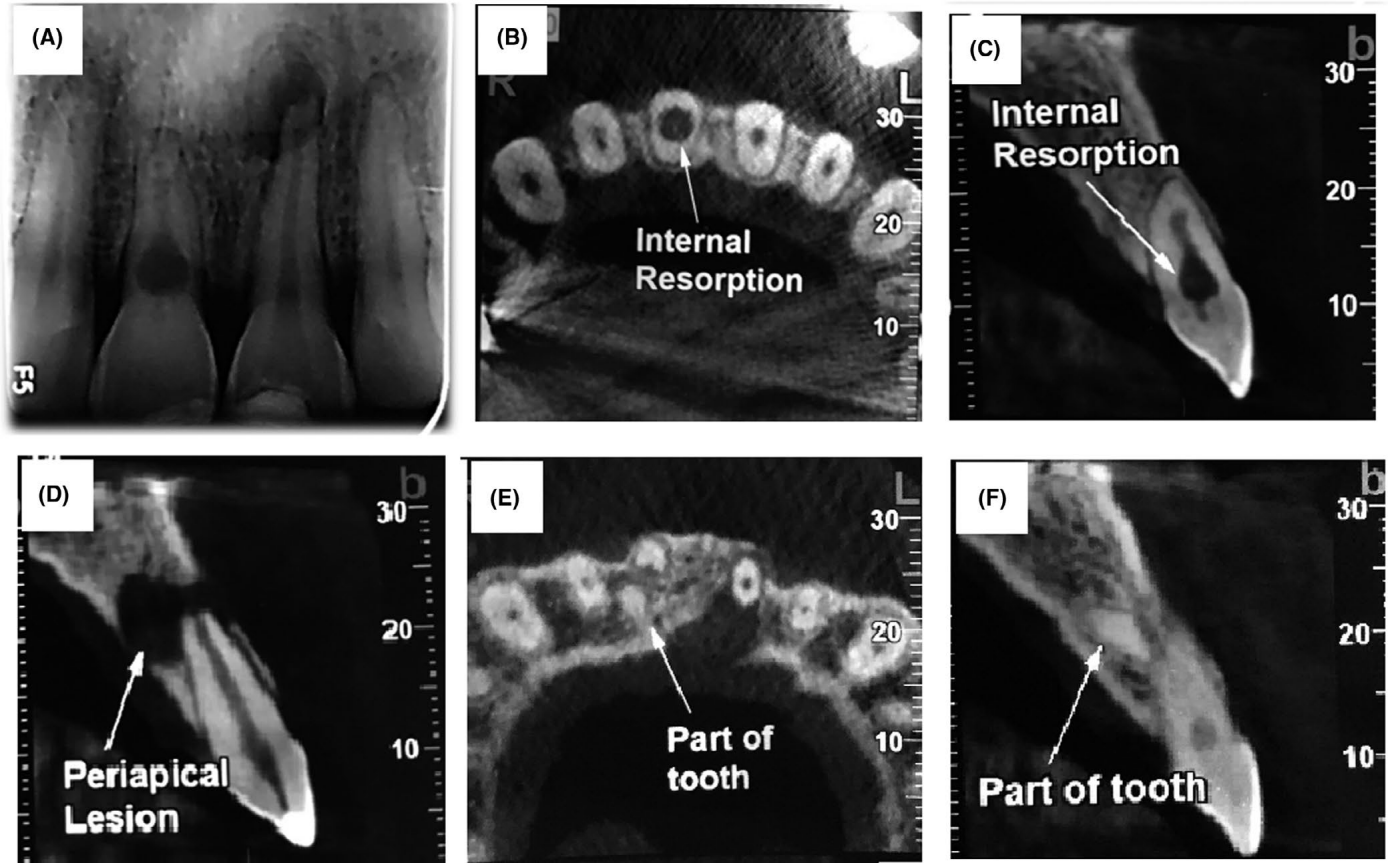
Preoperative radiography (A) and CBCT from axial (B, E) and sagittal view (C, D, F)

**FIGURE 3:**
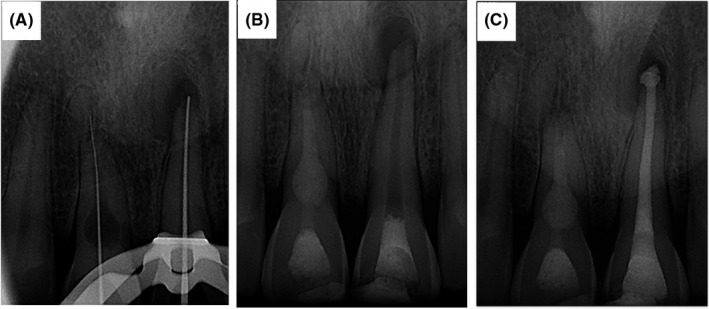
Periapical radiography showing working length determination (A), obturation of tooth #8 with Biodentine (B), and obturation of tooth #9 with MTA (C)

This was a case of a 40‐year‐old male patient referred from a general practitioner to the department of endodontics, QUMS, with the chief complaint of pain. The patient has had a trauma caused by accident 10 years ago. His medical history was unremarkable and classified as ASA I. In clinical evaluations, extraoral examination showed no facial asymmetry and swelling. Intraoral examination showed swelling in maxillary palate and sinus tract (Figure 1). The oral hygiene was found to be good. According to the pulp sensibility tests, teeth #8 and #9 did not respond. However, both of these teeth were tender to percussion/palpation. Intraoral periapical radiograph demonstrated periapical radiolucency and a leaking restoration in tooth #9 (Figure 2A). A dislocated fragment and root resorption with a uniform, radiolucent enlargement of the pulp canal was observed in tooth #8. CBCT was done (field of view 5 × 5), and axial, sagittal, and horizontal sections were obtained that aided in the diagnosis (Figure 2B‐D). Based on the clinical and radiographic findings, the patient was diagnosed with inflammatory internal root resorption (IRR) in tooth #8 alongside primary acute apical periodontitis. Tooth #9 had a pulpless and infected root canal system and was diagnosed with chronic apical abscess. Both teeth were subjected to RCT in the first visit. Before treatment, informed consent was taken from patient. After local anesthesia (2% lidocaine with 1/80000 Epinephrine; Daroupakhsh) and rubber dam isolation, fissure diamond bur and a high‐speed handpiece with water spray were used to prepare access cavity. The working length was determined by Root ZX electronic apex locator (J Morita MFQ) and confirmed with X‐ray (Figure 3A). Cleaning and shaping of the root canal system was completed using the Hybrid technique (hand & rotary instrument) followed by irrigation with normal saline and 5.25% NaOCl (master apical size #70). Canals were dried with sterile paper points and then the creamy Ca (OH)_2_ as an intracanal dressing was placed by lentulo. Finally, the access cavity was sealed temporarily with reinforced ZOE. The patient was recalled 1 month later. In the next visit, obturation was performed with Biodentine capsule (Septodont) for tooth #8 (Figure 3B). After local anesthesia with inferior alveolar nerve block (2% lidocaine with 1/80000 Epi), removal of the temporary filling materials followed by irrigation and drying was performed. Biodentine capsule was mixed with an amalgamator for 30 s at 4000 rpm, and after reaching a desired consistency, the resorption cavity was filled with Biodentine. After completion of root canal treatment, the tooth was restored using Glass Ionomer. Tooth #9 was obturated 3 days later in the third visit. After local anesthesia, removal of temporary materials, and irrigation and drying, placement of MTA by hand plugger for about 3 mm was performed and followed by removal of excess MTA by moistened paper points and restoration with reinforced ZOE. After 3 days, the patient was asymptomatic and the MTA setting was confirmed. Finally, obturation of tooth #9 was performed using Beefill thermoplasticized gutta‐percha technique (Figure 3C).

**FIGURE 4:**
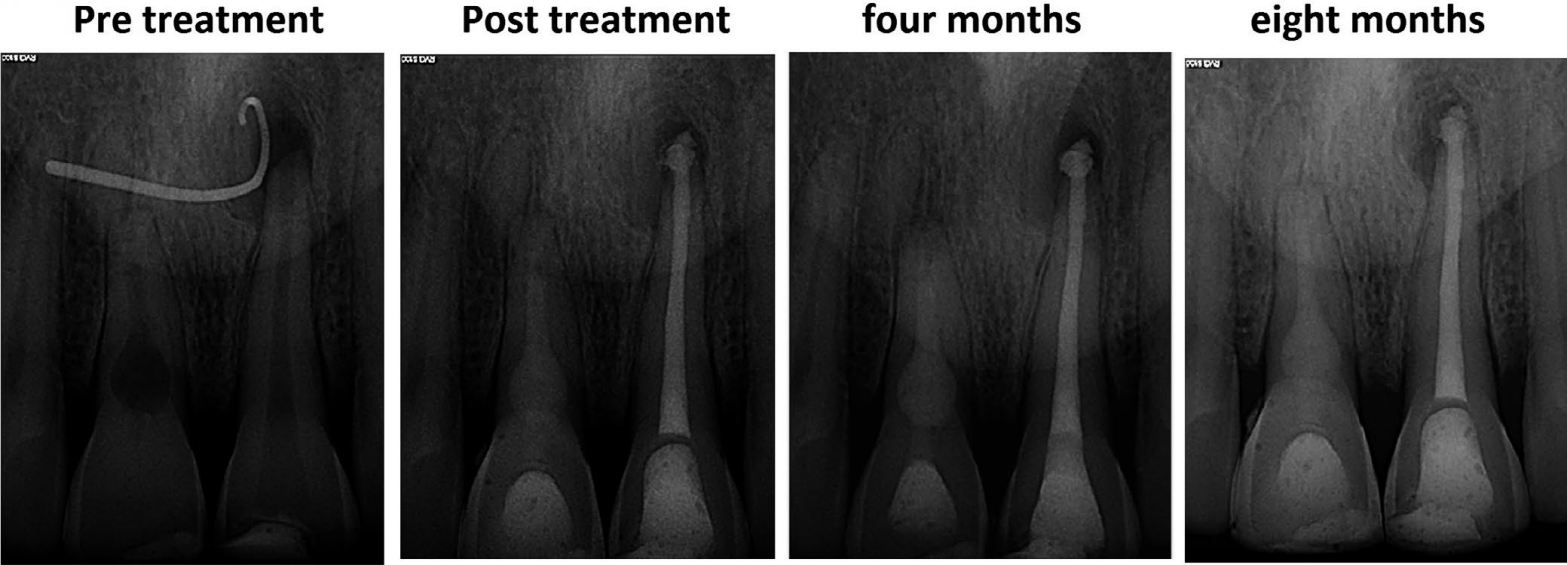
Pretreatment, post‐treatment, 3‐month, and 8‐month follow‐up radiographs

In the 3‐month and 8‐month follow‐up visit (Figure 4), tooth #8 was completely asymptomatic and did not represent any sign of further internal as well external resorptive progression of root structure. Tooth #9 did not show any tenderness to percussion/palpation and the periapical radiolucency was significantly reduced.

## 3 DISCUSSION

In endodontic discipline, there are many challenges about diagnosis and treatment of root resorption. Successful clinical outcome of such cases is determined by early and appropriate diagnosis of the root resorption. Differential diagnosis of IRR from other types of root resorptive defects is critical for tooth management and treatment planning by the clinician.^9^ The IRR may be misdiagnosed as external cervical resorption (ECR) in which the site of resorption is different. In contrast to ECR, an IRR cavity is continuous with the normal root canal walls because it is essentially an extension of them. In periapical radiographs, the canal walls should not be traceable through the resorptive defect in IRR. CBCT has overcome the limitations of conventional radiography and can demonstrate the correct type of resorption due to its capability to determine the exact location of the defect, its dimensions, and the presence of any wall perforations.^10^ In the present case, the IRR was confirmed with the CBCT that was used to locate the position and depth of the defect in relation to the root canal. Finally, CBCT was helpful to determine the restorability of the tooth and appropriate treatment planning.

In this case report, due to the thin and weakened structure in the resorptive defect of tooth #8, the commonly used bioactive materials, MTA or Biodentine, were suggested to be the candidates to reinforce the tooth and therefore improve the prognosis.^6^ Due to the superior characteristics, Biodentine was used as the obturating material for IRR due to its suitable consistency, one‐step obturation, better handling, and faster setting.^11^ Therefore, Biodentine is appropriate to effectively seal the resorptive cavity. Baranwal et al^12^ used Biodentine to manage invasive cervical resorption in maxillary left central incisor. In another study, root canal stenosis and external inflammatory resorption were managed by surgical root reconstruction using biodentine.^13^ Biodentine can be used as an effective alternative to MTA in various types of applications in Endodontics. Management of the tooth #8 with an open apex using Biodentine required significantly less time than other materials. It is clinically interesting, because the cleaning and shaping of the root canal is immediately followed by apical seal with a material that favors regeneration. In horizontal root fracture, the apical pulpal circulation is not disrupted so pulp necrosis is a rare phenomenon.^14^ In this case, since there was no complication associated with the apical segment of tooth #8 after 10 years, a clinical intervention was not performed. In this case, in tooth #9, periapical inflammation caused chronic apical abscess and apical resorption which caused an open apex. Therefore, the MTA apical plug was indicated.

## 4 CONCLUSION

The early differential diagnosis and appropriate treatment are critical steps to stop the resorptive process within the pulp canal space and improve the prognosis. In the present case, due to its desirable properties, Biodentine was successfully used to manage the IRR in the maxillary right central incisor with horizontal root fracture and dislocated apical segment. Simultaneously, the MTA apical plug backfilled with thermoplasticized gutta‐percha was successfully used to obturate the maxillary left central incisor with chronic apical abscess. After 3 months, successful healing and resolution of signs and symptoms were observed.

## DATA AVAILABILITY STATEMENT

No datasets were generated or analyzed during this case report.

## CONFLICT OF INTEREST

None declared.

## AUTHOR CONTRIBUTIONS

PS: reviewed the literature, developed the concept and design of the study, performed the procedure, and drafted the manuscript. MB: reviewed the literature, involved in data analysis/interpretation, and drafted the manuscript. FN: involved in concept/design, analysis, and data collection. EM: involved in concept/design and revised the manuscript.

## ETHICAL APPROVAL

This case report meets the ethical guidelines and adheres to Iran's local legal requirements.

## CONSENT STATEMENT

Published with written consent of the patient.
